# Development the Care Evaluation Scale Version 2.0: a modified version of a measure for bereaved family members to evaluate the structure and process of palliative care for cancer patient

**DOI:** 10.1186/s12904-017-0183-2

**Published:** 2017-01-23

**Authors:** Mitsunori Miyashita, Maho Aoyama, Misato Nakahata, Yuji Yamada, Mutsumi Abe, Kazuhiro Yanagihara, Akemi Shirado, Mariko Shutoh, Yoshiaki Okamoto, Jun Hamano, Aoi Miyamoto, Saki Yoshida, Kazuki Sato, Kei Hirai, Tatsuya Morita

**Affiliations:** 1 Department of Palliative Nursing, Health Sciences, Tohoku University Graduate School of Medicine, 2-1 Seiryo-machi, Aoba-ku, 980-8575 Sendai Japan; 2 Aiwa Hospital, Nagano, Japan; 3 Department of Palliative Care and Pain Clinic, Matsue City Hospital, Matsue, Japan; 4 Department of Medical Oncology, Kansai Electric Power Hospital, Osaka, Japan; 5 Department of Palliative and Supportive Care, Palliative Care Team and Seirei Hospice, Seirei Mikatahara Hospital, Hamamatsu, Japan; 6 Department of Palliative Medicine, Oita City Medical Association’s Almeida Memorial Hospital, Oita, Japan; 7 Wata Clinic, Tokyo, Japan; 8 Department of Pharmacy, Ashiya Municipal Hospital, Ashiya, Japan; 9 Division of Clinical Medicine, Faculty of Medicine, University of Tsukuba, Tsukuba, Japan; 10 Institute of Academic Initiatives, & Graduate School of Human Sciences, Osaka University, Osaka, Japan; 11 Department of Palliative and Supportive Care, Palliative Care Team and Seirei Hospice, Seirei Mikatahara Hospital, Shizuoka, Japan

**Keywords:** Palliative care, Quality of care, Neoplasms, Questionnaires, Bereavement

## Abstract

**Background:**

The Care Evaluation Scale (CES1.0) was designed to allow bereaved family members to evaluate the structure and process of care, but has been associated with a high frequency of misresponses. The objective of this study was to develop a modified version of CES1.0 (CES2.0) that would eliminate misresponses while maintaining good reliability and validity.

**Methods:**

We conducted a cross-sectional questionnaire survey by mail in October 2013. The participants were bereaved family members of patients who died from cancer in seven institutions in Japan. All family members were asked to complete CES2.0, the short form CES1.0, items on overall care satisfaction, the Family Satisfaction with Advanced Cancer Care (FAMCARE) Scale, the Patient Health Questionnaire-9 (PHQ-9) and the Brief Grief Questionnaire (BGQ). To examine test-retest reliability, all participants were asked to complete a second CES2.0.

**Results:**

Of 596 questionnaires sent, 461 (77%) were returned and 393 (66%) were analyzed. In the short form CES1.0, 17.1% of the responses were identified as misresponses. No misresponses were found in CES2.0. We identified 10 CES2.0 subscales similar to those in CES1.0 using exploratory factor analysis. Cronbach’s alpha was 0.96, and the intraclass correlation coefficient was 0.83. Correlations were found between CES2.0 and overall satisfaction (*r* = 0.83) and FAMCARE (*r* = 0.58). In addition, total CES2.0 scores were negatively correlated with the PHQ-9 (*r* = −0.22) and BGQ (*r* = −0.10).

**Conclusion:**

These results suggest that CES2.0 eliminated misresponses associated with CES1.0 while maintaining good reliability and validity and greatly improving test-retest reliability.

## Background

The way in which the quality of medical care is measured is an essential part of accurate quality assurance. In palliative care, in addition to the difficulties associated with prognostication, patients are frequently too ill to complete questionnaire surveys or take part in interviews. Therefore, it is common to have bereaved family members evaluate the provision of care at the patients’ end-of life [1].

Donabedian [2] stated that quality of care consists of the following three components: 1) structure of care; 2) process of care; and 3) outcome of care. Although numerous measures have been developed to evaluate the outcomes of palliative care, such as quality of the dying process [3–7], few measures have been developed to evaluate the structure and process of care [3]. One of these measures is the Care Evaluation Scale (CES), which was developed in 2004 to evaluate the palliative care structure and process [8]. The CES has been used in a number of nationwide surveys and clinical audits by institutions in Japan [9–11].

To measure the structure and process of care as experienced by bereaved family members, the original CES (CES1.0) consists of 28 items with the following 10 subscales: physical care by physicians; physical care by nurses; psycho-existential care; help with decision-making for patients; help with decision-making for the family; environment; family burden; cost; availability; and coordination/consistency. In CES1.0, bereaved family members are asked whether improvements are needed in regard to the care provider. This is because when CES1.0 was developed, the provision of care was typically rated using agreement or satisfaction level, but this makes it difficult to interpret whether the respondents actually desire further improvement in this area [8].

However, during data entry, we noticed that the care assessment method used in CES1.0 appeared to result in misresponses by a substantial proportion of the respondents. In this article, we define misresponse operationally that “misresponse occurs when a respondent selects an option that is inconsistent or incongruent with his or her corresponding beliefs”. For example, respondents who described themselves as being “very satisfied” with the provision of care also responded that “improvement is highly necessary”. We assume that such respondents misunderstood “highly necessary” as “the item, for example physical care by the physician, is very good”. Although this may be somewhat confusing to English readers, the wording of the item in Japanese was thought to lead to reverse-score errors in the responses if not read carefully. In Japanese, although the question regarding satisfaction was quite straightforward, the question regarding the necessity of improvement was in the form of a double negative, and therefore somewhat confusing. In fact, we found that question regarding satisfaction was consistent with the other questions, while the question regarding the necessity of improvement was consistent with those associated with suspected misresponses. Consequently, we found a misresponse rate of 5–10% (unpublished data). Therefore, we referred to the overall satisfaction question in previous studies and manually corrected the inverse responses to reflect the inverse score [9–11]. However, this was not only time consuming, but also insufficient, because categorizing respondents who answer “somewhat satisfied” for the overall satisfaction question as satisfied or unsatisfied, which would be necessary to correct such CES1.0 responses, was not feasible. Alternatively, we hypothesized that we could eliminate misresponses by improving the language of the response options on the questionnaire. Therefore, the objective of this study was to develop a modified version of CES1.0 (CES2.0) that would allow the quality, structure, and process of palliative care to be evaluated more accurately while maintaining good reliability and validity.

## Methods

### Participants and procedures

We conducted a cross-sectional questionnaire survey by mail in October 2013. The potential participants were bereaved family members of 100 consecutive patients who died in four inpatient palliative care units, two home hospices, and a general hospital ward before May 31, 2013. Patients who died before 2012 were not included because a similar bereavement study in a palliative care unit was conducted in 2013. The inclusion criteria were as follows: 1) the patient died from cancer; and 2) the patient and bereaved were at least 20 years of age. The exclusion criteria were as follows: 1) the responsible family member or guarantor could not be identified; 2) treatment-related death or death in the intensive care unit; 3) the bereaved had psychological distress at level that kept them from participating in the study as determined by the primary physician; and 4) the bereaved was incapable of completing the questionnaire due to cognitive dysfunction or the inability to read Japanese. We estimated that a sample size of 200 would be required based on exploratory factor analysis, convergent validity, and test-retest reliability [12].

We sent a questionnaire by mail to the responsible family member or guarantor as noted in the hospital records. They were asked to complete the questionnaires by the primary caregiver of the deceased. Reminders were sent to all non-responders 2 weeks later. This retest interval was standard in Japan and was also elected in acoordance with development of patient version of CES [13]. Potential participants who refused to participate were asked to indicate their decision in a check box on the cover sheet and return the questionnaire. Reminders were not sent to such responders. To examine the test-retest reliability of CES2.0, a retest questionnaire was sent to all respondents 2 weeks after they returned the first questionnaire. In Japan, written informed consent is not required for anonymous questionnaire surveys. Potential participants were informed about the details of the survey, and completing and returning a questionnaire was regarded as voluntary consent to participate.

This study was approved by the institutional review boards of Tohoku University and all participating institutions and conducted in accordance with the ethical guidelines for epidemiological research issued by the Ministry of Education, Culture, Sports, Science, and Technology and the Ministry of Health, Labour, and Welfare of Japan.

### Measurements

#### Care Evaluation Scale version 2.0 (CES2.0)

We developed CES2.0 based on modifications from the original CES1.0 for bereaved family members [8]. In CES1.0, participants were asked whether improvements were necessary in relation to the care provider. Because this appeared to be confusing for the participants, we changed the response option to a 6-point Likert scale (6: highly agree; 5: agree; 4: somewhat agree; 3: somewhat disagree; 2: disagree; 1: highly disagree). We also asked participants to select “7: N/A” if none of the other scores were applicable to the patient.

CES1.0 consisted of 28 items with 10 subscales. We modified the expression of each item to match the new response options without changing the underlying concept of each subscale. For example, the statement “The same doctors and nurses provided care” in CES1.0 was changed to “Important information was shared even when the attending physician or nurse was changed” in CES2.0. To allow easy interpretation, all scores were proportionally adjusted to range from 0 to 100, similar to CES1.0; higher scores indicated good structure or process of care. We designed this scale to be used not only in inpatient settings, but also in the home.

#### Short version of the original Care Evaluation Scale (CES1.0)

We used the short version of the original CES1.0 [8], which consists of 10 items and 10 subscales. In the original CES1.0, participants were asked to indicate if improvements were necessary using the following 6-point Likert scale (“1: improvement is not necessary”; “2: improvement is little necessary”; “3: improvement is somewhat necessary”; “4: improvement is necessary”; “5: improvement is quite necessary”; and “6: improvement is highly necessary”).

#### Overall care satisfaction

Participants were asked about their overall satisfaction with care using the following question: “Overall, in the past month, were you satisfied with the medical care the patient received in the last place of care?” Participants were asked to respond on a 6-point Likert scale (“1: highly dissatisfied” to “6: highly satisfied”). This measure was used in the development of CES1.0 [8] and in a nationwide bereavement study [11].

#### FAMCARE (Family Satisfaction with Advanced Cancer Care)

We used the FAMCARE Scale to measure family satisfaction with advanced cancer care. The original FAMCARE Scale is composed of 20 items that can be rated on a 7-point Likert scale (“7: highly satisfied” to “1: highly dissatisfied”) [14]. The Japanese version of the FAMCARE Scale was translated by the forward and backward translation procedure [15].

#### Expectation of care

Expectation of care before receiving the last place of care was also investigated using a method similar to that for CES2.0. Participants were asked to rate their level of expectation for seven items (physical care by physician; physical care by nurse; psycho-existential care; help with decision-making; environment; family burden; and cost) on a 3-point Likert scale (“1: not very expected” to “3: highly expected”) [8].

#### Patient Health Questionnaire-9 (PHQ-9)

We used the Patient Health Questionnaire-9 (PHQ-9) to measure depression among the participants. The PHQ-9 is a self-administered questionnaire which scores each of the nine Diagnostic and Statistical Manual of Mental Disorders version IV criteria for depression from “0” (not at all) to “3” (nearly every day) [16]. We used the Japanese version of the PHQ-9, which has confirmed validity [17].

#### Brief Grief Questionnaire

We used the Brief Grief Questionnaire (BGQ) to measure the grief of the participants. The original BGQ was developed for a study on the 9–11 terrorist attacks in New York City. It is composed of five items that can be rated on a 3-point Likert scale, and can be easily administered [18]. The validity of the Japanese version of the BGQ was also confirmed in the general Japanese population [19].

#### Participants’ characteristics

Information on the patients’ age, sex, primary cancer site, month after death, place of death, and duration of stay in the last place of care (hospital or home) was collected from medical charts, while data on family age, sex, relationship to the deceased, physical and mental health status during the caregiving period, frequency attending to the patient, presence of other caregivers, education, medical expenditures during the last month, annual household income during the caregiving period, and feelings regarding the household budget during the caregiving period were collected by questionnaire.

Although CES2.0, overall care satisfaction, and the PHQ-9 were administered to all participants, in consideration of the volume of the questionnaire, CES1.0, the FAMCARE Scale, the BGQ and expectation of care were only administered to half of the participants, and the retest questionnaire only consisted of CES2.0.

### Analysis

Before analysis, we identified the likely misresponses on CES2.0 and the short version of the CES1.0 by referring to the question on overall satisfaction. We then counted and manually corrected the misresponses. We calculated descriptive statistics and conducted item analysis. We treated the response “7: N/A” on CES2.0 as a missing value. To calculate the subscale scores, we imputed mean responses from within each subscale for all missing values.

We tested the reliability of the factor structure using the split-half method; we randomly split the data in half and performed exploratory factor analysis on one half, and confirmatory factor analysis on the other half. For internal consistency and test-retest reliability, we calculated Cronbach’s alpha coefficients and intraclass correlation coefficients (ICC). To examine concurrent and discriminant validity, we calculated Pearson’s correlation coefficients between the total score and each subscale score for CES2.0, overall satisfaction, the FAMCARE Scale, expectation of care, the PHQ-9 and the BGQ. In addition, we calculated Pearson's correlation coefficients between CES2.0 and the short version of CES1.0. Finally, we developed a short version of CES2.0 by selecting one item from all subscales. The selection criteria were as follows: 1) high correlation with subscale total score (*r* > 0.90); and 2) high reliability as a single item (ICC > 0.60) and low missing rate (<10%). All analyses were performed using the SAS statistical package (version 9.4; SAS Institute, Cary, NC).

## Results

### Participant characteristics

We identified 692 potential participants in six facilities. Among these, 92 bereaved were excluded for the following reasons: 1) could not identify the responsible family member or guarantor (*n* = 41, 45%); 2) treatment-related death or death in intensive care unit (*n* = 8, 9%); 3) serious psychological distress (*n* = 23, 25%); 4) incapable of completing the questionnaire (*n* = 2, 2%); and 5) other (*n* = 14, 15%). A total of 600 questionnaires were mailed, four of which were returned due to a wrong address. Of 596 questionnaires successfully sent, 461 (77%) responses were received; among these, 68 were refusals to participate in the study. The reasons for refusal were as follows: “It is too emotionally burdensome to recall the patient's death” (*n* = 33, 49%); “Please leave me in peace” (*n* = 26, 37%); “The hospitalized period was too short to evaluate care” (*n* = 21, 31%); “I am not good at answering questionnaires” (*n* = 13, 19%); and others (multiple answers). Finally, 393 (66%) valid questionnaires were analyzed. To examine test-retest reliability, we sent 393 questionnaires, from which 219 (56%) were returned.

The participants’ characteristics are shown in Table 1. The mean patient age ± standard deviation (SD) was 73 ± 12 years, and males made up 60% of the total. The place of death was as follows: inpatient palliative care units (53%); home (32%); and general hospital ward (15%). The mean duration of stay in the last place of care (hospital or home) was 52 ± 90 days, and time after death was 14 ± 8 months. The mean family age was 61 ± 12 years, and females made up 69% of the total. Spouses comprised 50% and children 35% of the family members. A total of 84 family members attended to the patient 4 days or more per week. No significant differences of characteristics collected for lists were observed between responders and non-responders.

**Table 1:** Participants’ characteristics

	Number	Percent
Patient		
Age, y, mean ± SD (median, range)	73.2 ± 12.1	(75, 32–101)
Sex		
Male	235	60
Female	156	40
Primary site		
Lung	93	24
Stomach/Esophagus	57	15
Colon/Rectum	51	13
Liver/Gallbladder/Bile duct	36	9
Urinary	33	8
Pancreas	31	8
Uterus/Ovarian	20	5
Leukemia/Malignant lymphoma	15	4
Head and neck	12	3
Breast	8	2
Other	37	9
Place of death		
Palliative care unit	208	53
Home	127	32
Hospital general ward	58	15
Hospital or home days, mean ± SD (median, range)	52.2 ± 90.0	(27, 0–1040)
Months after death, mean ± SD (median, range)	14.2 ± 7.6	(12.0, 5.0–45.4)
Family		
Family age, y, mean ± SD (median, range)	61.4 ± 12.1	(62, 27–94)
Family sex		
Male	118	30
Female	273	69
Relationship to decedent		
Spouse	198	50
Child	138	35
Child-in-law	30	8
Parent	16	4
Sibling	6	2
Other	4	1
Physical health status during the caregiving period		
Good	113	29
Moderate	197	50
Fair	58	15
Bad	15	4
Mental health status during the caregiving period		
Good	61	16
Moderate	179	46
Fair	115	29
Bad	28	7
Frequency of attending to the patient		
Every day	285	73
4–6 days/week	45	11
1–3 days/week	37	9
less than 1 day/week	21	5
Presence of other caregivers		
Present	282	72
Absent	105	27
Education		
Junior high school or less	33	8
High school	183	47
College	103	26
University/Graduate school	63	16
Other	5	1
Medical expenditure during the last month (thousand yen)^a^		
≤99	104	26
100–199	121	31
200–399	100	25
400–599	33	8
≥600	16	4
Household annual income during the caregiving period (thousand yen)		
≤999	27	7
1000–1999	74	19
2000–3999	136	35
4000–5999	68	17
6000–7999	40	10
≥8000	31	8
Feelings regarding the household budget during the caregiving period		
Difficult	26	7
Somewhat difficult	58	15
No problem	206	52
Somewhat affluent	60	15
Affluent	38	10

Some totals are not 100% due to missing values

^a^On average, 100,000 yen was equal to 1,025 USD in 2013

### Item analysis and factor validity

In the short version of CES1.0, we found a misresponse rate of 17.1%; in contrast, no misresponses were found on CES2.0. The results of the item analysis with all data, and the factor analysis with one half of the split data are shown in Table 2. We succeeded in re-creating the factor structure of CES1.0, with confirmatory factor analysis on the other half of the split data [chi-square = 736.7 (df = 338), *P* = 0.000; CFI = 0.91, TLI = 0.90, RMSEA = 0.092, SRMR = 0.067].

**Table 2:** Item and factor analysis

	Standardized regression coefficients										Communarity	Missing value (%)	Pearson's r ^b^	ICC ^c^
	F1	F2	F3	F4	F5	F6	F7	F8	F9	F10				
V. Explanation to family by phsician (mean = 82, SD = 17)														
13. Physician gave sufficient explanation to the family about condition and the medical treatment.^a^	**0.95**	−0.10	−0.03	0.01	0.06	−0.01	0.08	−0.02	0.02	0.00	0.92	2.03	0.95	0.76
14. Physician gave easy-to-understand explanation to the family about condition and the medical treatment.	**0.91**	0.00	−0.01	−0.03	0.03	−0.05	0.10	−0.01	−0.02	0.11	0.94	2.28	0.96	0.70
15. Consideration is given so that the family would participate in the selection of treatment.	**0.86**	0.08	0.05	−0.05	0.04	−0.08	0.03	0.09	0.06	−0.02	0.91	2.54	0.94	0.75
III. Pycho-existential care (mean = 84, SD = 16)														
7. Physicans, nurses and staff endevered to relieve patient’s concerns and worries.^a^	0.09	**0.74**	0.00	0.04	0.06	−0.16	0.22	0.03	0.03	0.06	0.90	2.79	0.96	0.69
8. Physicans, nurses and staff endevored to relieve patient’s sadness and depression.	−0.03	**0.84**	−0.02	0.07	0.07	0.02	0.06	−0.04	−0.01	0.10	0.93	3.55	0.96	0.69
9. Physicans, nurses and staff endevered so that the patient’s hope would be accomplished.	0.12	**0.83**	0.04	0.04	0.04	0.12	−0.09	−0.05	0.00	−0.03	0.91	2.79	0.93	0.64
IX. Availability (mean = 82, SD = 17)														
23. Admission (use) is possible when necessary without waiting.^a^	0.01	0.13	**0.95**	−0.01	−0.10	0.07	−0.06	0.02	−0.08	−0.10	0.88	8.12	0.90	0.70
24. The procedures of admission (use) are simple.	0.02	−0.02	**0.89**	−0.03	0.08	−0.05	0.02	0.01	0.03	−0.03	0.85	8.38	0.89	0.60
25. Admission (use) is in accordance with the wishes of the patient and family.	−0.01	−0.14	**0.83**	0.01	0.09	−0.05	0.14	−0.01	0.06	0.18	0.86	8.88	0.88	0.66
VI. Environment (mean = 78, SD = 20)														
16. Hospital or room was convenient and comfortable.^a^	−0.02	0.05	−0.02	**0.82**	0.07	0.01	0.09	−0.19	0.15	0.04	0.87	1.27	0.92	0.60
17. Environment was quiet and calm .	−0.03	−0.10	−0.05	**0.87**	0.09	−0.11	0.04	0.23	−0.08	0.14	0.90	3.55	0.89	0.59
18. Toilet and washstand facilities was convinient.	0.00	0.13	0.04	**0.91**	−0.12	0.10	−0.05	0.07	0.00	−0.16	0.87	5.84	0.90	0.74
X. Coordination and consistency (mean = 80, SD = 17)														
26. There is good cooperation among staff members such as physicians and nurses.^a^	−0.08	0.19	0.07	−0.06	**0.80**	0.03	−0.02	0.07	0.01	0.04	0.90	2.03	0.93	0.72
27. Important information was shared even when the attending physician or nurse was changed.	0.03	0.07	−0.03	−0.01	**0.93**	−0.03	0.11	−0.03	−0.02	−0.06	0.94	3.30	0.95	0.72
28. Treatment is planned with appropriate consideration of the previous course of the disease.	0.23	−0.06	0.07	0.09	**0.81**	0.14	−0.16	−0.04	−0.01	−0.06	0.92	4.31	0.92	0.72
I. Physical care by physician (mean = 85, SD = 15)														
1. Physicans endevered to relieve physical discomfort of the patient^a^	0.08	−0.01	−0.02	0.02	0.06	**0.77**	0.17	0.08	0.01	−0.04	0.91	1.27	0.93	0.70
2. Physicans deal promptly with discomforting physical symptoms of the patient	−0.05	−0.03	0.04	0.00	0.04	**0.74**	0.14	−0.02	0.03	0.24	0.88	2.79	0.93	0.67
3. Physicans have adequate knowledge and skills to alleviate physical symptoms of the patient	0.18	0.14	−0.04	−0.01	0.04	**0.67**	0.11	0.01	−0.02	0.00	0.89	2.79	0.91	0.71
II. Physical care by nurse (mean = 83, SD = 18)														
4. Nurses endevered to relieve physical discomfort of the patient^a^	0.12	−0.07	0.01	0.10	−0.04	0.26	**0.83**	−0.07	−0.04	−0.11	0.92	3.81	0.92	0.64
5. Nurses deal promptly with discomforting physical symptoms of the patient	0.00	0.32	0.12	0.05	−0.05	−0.01	**0.73**	−0.02	−0.02	0.03	0.93	3.55	0.93	0.70
6. Nurses have adequate knowledge and skills to alleviate physical symptoms of the patient	−0.05	0.38	−0.11	−0.18	0.11	0.16	**0.49**	0.16	0.07	−0.03	0.82	3.30	0.91	0.70
VIII. Consideration with family health (mean = 77, SD = 16)														
21. Consideration was given to the health of family^a^	−0.01	−0.09	0.03	−0.02	0.00	0.11	−0.04	**0.86**	0.12	0.07	0.89	3.81	0.94	0.59
22. Consideration was given so that the family could have their own time and continue to work	0.05	0.07	0.00	0.10	−0.01	−0.05	0.02	**0.93**	−0.05	−0.04	0.91	5.33	0.94	0.50
VII. Cost (mean = 74, SD = 19)														
19. The total cost is reasonable.^a^	0.04	0.10	−0.06	0.06	0.00	−0.06	0.04	−0.02	**0.89**	0.02	0.93	7.11	0.95	0.60
20. The contents of the bills are easy to understand.	0.02	−0.05	0.06	0.01	−0.01	0.07	−0.07	0.08	**0.94**	−0.06	0.93	10.41	0.95	0.65
IV. Explanation to patient by phsician (mean = 80, SD = 18)														
10. Physician gave sufficient explanation to the patient about condition and the medical treatment.^a^	0.38	0.25	0.01	−0.03	−0.08	0.12	−0.11	−0.02	0.04	**0.59**	0.93	3.55	0.94	0.68
11. Physician gave easy-to-understand explanation to the patient about condition and the medical treatment.	0.41	0.11	−0.03	0.01	−0.02	0.12	−0.03	0.07	−0.07	**0.63**	0.95	2.03	0.96	0.67
12. Consideration is given so that the patient would participate in the selection of treatment.	0.26	0.14	0.09	0.05	−0.04	0.22	−0.10	−0.03	0.01	**0.55**	0.84	5.58	0.90	0.70

^a^items of short version

^b^Pearson’s correlation coefficient with each subscale total score

^c^intraclass correlation coefficient

Missing values ranged from 1.3% to 10.4%. The ICCs of all but one item were over 0.60 and we retained this item because it was considered indispensable and the ICC was 0.59.

We identified 10 subscales similar to CES1.0 using exploratory factor analysis. The mean ± SD total score on CES2.0 was 81.0 ± 12.4. The CES1.0 short version items are indicated with asterisks in Table 2.

### Internal consistency and reliability

Internal consistency (Cronbach’s alpha) and test-retest reliability (ICC) are shown in Table 3. Cronbach’s alpha ranged from 0.87 to 0.95 (total score: 0.96; short version: 0.89), and ICC ranged from 0.56 to 0.77 (total score: 0.83; short version 0.82).

**Table 3:** Internal consistency and reliability

Domain	Alpha	ICC
I. Physical care by physician	0.92	0.76
II. Physical care by nurse	0.91	0.76
III. Pycho-existential care	0.95	0.73
IV. Explanation to patient by physician	0.93	0.68
V. Explanation to family by physician	0.95	0.77
VI. Environment	0.89	0.57
VII. Cost	0.89	0.65
VIII. Consideration of family health	0.87	0.56
IX. Availability	0.87	0.75
X. Coordination and consistency	0.93	0.75
Total score	0.96	0.83
Total score (short version)	0.89	0.82

alpha: Cronbach’s alpha coefficient

ICC: Intraclass correlation coefficient

### Concurrent and discriminant validity

Concurrent and discriminant validity as demonstrated by Pearson’s correlation coefficient are shown in Table 4. The CES2.0 total score was correlated with overall satisfaction (*r* = 0.83) and FAMCARE (*r* = 0.58), but not with expectation of care (*r* = 0.02). In addition, the CES2.0 total score was negatively correlated with the PHQ-9 (*r* = −0.22) and the BGQ (*r* = −0.10). Although these results were similar to those found using the short version of CES1.0, CES2.0 was more strongly correlated with overall satisfaction (*r* = 0.71) and FAMCARE (*r* = 0.53).

**Table 4:** Concurrent/discriminant validity of CES2.0 and the short version of CES1.0

CES2.0							
Domain	Overall satisfaction	FAMCARE	Expectation	PHQ-9	BGQ	CES1.0 (domain)^a^	CES1.0 (item)^b^
I. Physical care by physician	0.70	0.50	0.05	−0.21	−0.06	0.59	0.53
II. Physical care by nurse	0.69	0.44	0.09	−0.18	−0.08	0.68	0.62
III. Pycho-existential care	0.70	0.44	0.01	−0.15	−0.06	0.71	0.67
IV. Physician’s explanation to the patient	0.64	0.50	−0.07	−0.18	0.01	0.66	0.63
V. Physician’s explanation to the family	0.67	0.53	0.04	−0.22	−0.02	0.68	0.65
VI. Environment	0.46	0.29	0.02	−0.15	−0.10	0.63	0.66
VII. Cost	0.62	0.47	−0.01	−0.15	−0.21	0.55	0.57
VIII. Consideration of family health	0.46	0.30	0.17	−0.15	−0.09	0.56	0.55
IX. Availability	0.54	0.45	−0.01	−0.13	−0.04	0.62	0.68
X. Coordination and consistency	0.79	0.51	−0.05	−0.16	−0.20	0.68	0.64
Total score	0.83	0.58	0.02	−0.22	−0.10	0.78	-
Total score(short version)	0.82	0.59	0.02	−0.20	−0.12	0.79	-
Short version of the CES1.0							
I. Physical care by physician	0.63	0.50	0.02	−0.17	-	-	-
II. Physical care by nurse	0.63	0.41	0.00	−0.21	-	-	-
III. Pycho-existential care	0.59	0.44	0.00	−0.16	-	-	-
IV. Physician’s explanation to the patient	0.55	0.47	−0.07	−0.20	-	-	-
V. Physician’s explanation to the family	0.54	0.51	−0.09	−0.20	-	-	-
VI. Environment	0.54	0.30	−0.11	−0.25	-	-	-
VII. Cost	0.49	0.33	−0.01	−0.22	-	-	-
VIII. Consideration of family health	0.57	0.37	0.03	−0.18	-	-	-
IX. Availability	0.47	0.42	−0.03	−0.08	-	-	-
X. Coordination and consistency	0.61	0.45	−0.02	−0.21	-	-	-
Total score	0.71	0.53	−0.04	−0.24	-	-	-

Values indicate Pearson correlation coefficients. We could not analyze correlation of BGQ and CES1.0 because of combination of questionnaire

PHQ-9: Patient Health Questionnaire. BGQ: Brief Grief Questionnaire

^a^Correlation coefficient between domain of CES2.0 and corresponding item of short version of the CES1.0

^b^Correlation coefficient between item of short version of CES2.0 and corresponding item of short version of the CES1.0

### Correlation between CES2.0 and CES1.0

We added correlation between CES2.0 and CES1.0 in Table 4. The correlation coefficient between CES2.0 and CES1.0 total scores was 0.78 and that between the corresponding subscales ranged from 0.56 to 0.71. In addition, as more comparable case, the correlation coefficient of CES2.0 total score of short version and CES1.0 was 0.79 and that between the corresponding items ranged from 0.53 to 0.67.

## Discussion

CES2.0 was shown to have good reliability and validity. The most important result was that the misresponse rate was 0% in CES2.0, in contrast to 17% in CES1.0. In addition, no decrease in validity was seen in CES2.0 compared with CES1.0, and test-retest reliability was greatly improved.

Regarding the misresponse rate, in this study, 17% of the respondents incorrectly responded to items on the short version of CES1.0. This rate was higher than that found in our past experiences (from 5–10%). The respondents might have been less careful in answering items on CES1.0 in this study than in previous studies because they were asked to complete similar questionnaires (CES2.0 and CES1.0), and CES1.0 was administered after the other measures.

The test-retest reliability of CES2.0 was high (total score: *ρ* = 0.83). In another study involving CES1.0, the test-retest reliability was reported as 0.57 [8]. We believe that this improved reliability can be explained as follows. First, CES2.0 was easier to understand that CES1.0 and no misresponses were identified. Second, the test-retest interval of the previous study was 6 months [8], which was much longer than that used in this study. In developing CES2.0, several expressions for questionnaire items were modified to be easier to answer without altering the concept of the subscale. Therefore, respondents could respond to items more accurately.

No other psychometrics of CES2.0 decreased after the modifications from CES1.0. Factor validity, internal consistency, and concurrent/discriminant validity were all good. The correlations between CES2.0 and overall care satisfaction and the FAMCARE Scale were slightly higher than those with CES1.0. This could also be the result of the elimination of misresponses from CES2.0.

In developing CES2.0, we did not adopt satisfaction as a measurement concept because this was often criticized as being insufficiently theorized and having no widely accepted definition [20]. One of the criticisms of the satisfaction measure is the effect of expectation of care in studies in Australia and the US [21–23]. In this study, CES2.0 was not correlated with expectation of care, and slightly correlated with depression among the bereaved. This slight correlation is consistent with that observed with CES1.0 in this study, the original CES1.0 study, and other previous studies [8, 24]. It is therefore difficult to make a causal inference between depression and satisfaction because lower satisfaction of care might lead to depression in bereaved family members [25]. In this study, because only a slight correlation was observed between CES2.0 and depression or grief, this was not thought to have had a large effect on the perception of quality of care among the bereaved.

This study did have several limitations. First, as described in the Analysis section, misresponses were checked manually, which is an imperfect procedure. It is possible that some misresponses may have gone undetected in CES2.0. Second, 85% of the patients died in inpatient palliative care units or home hospices. However, in Japan, the actual proportion of patients who die in these places of care is less than 20%. Therefore, our study sample may not be representative of the general population. It may be better to use descriptive statistics based on place of care because the quality of care in inpatient palliative care units and home hospices is more highly rated than general hospital wards in Japan [11]. Because of the possibility of a ceiling effect, an additional survey using a more representative sample might be necessary to confirm the factor structure and achieve high convergent validity.

Third, the ceiling effect of responses might have resulted in an underestimation of validation data. Fourth, we compared CES2.0 to the short version of CES1.0 instead of the long version due to space limitations. A comparison between the CES2.0 and the CES1.0 long forms might provide more accurate results. This is because the space of questionnaire was limited because this survey had several objectives simultaneously and contained more questions other than development of the CES2.0. Fifth, in this study, we did not conduct cognitive interviews with the participants. Performing cognitive interviews could help provide important respondent feedback on the language, comprehensibility, ambiguity, and relevance of the items. Sixth, due to ethical considerations, bereaved family members considered to have severe psychological distress as determined by the primary physician were excluded. This might have resulted in a selection bias. Finally, the possibility of additional biases, including a recall bias, a cultural bias (this survey was only conducted in a Japanese population), and a bias in that this scale was only evaluated in relation to cancer patients, cannot be excluded.

## Conclusions

The modified CES2.0 demonstrated good reliability and validity. The most important result was that the misresponse rate in CES2.0 was 0%, in contrast to the 17% in CES1.0 found in this study. Second, no other CES2.0 psychometrics decreased after the modifications from CES1.0. Furthermore, test-retest reliability was greatly improved.

## Abbreviations

BGQ: Brief Grief Questionnaire
CES: Care Evaluation Scale
FAMCARE: Family Satisfaction with Advanced Cancer Care
ICC: Intraclass correlation coefficient
PHQ-9: Patient Health Questionnaire-9
SD: Standard deviation
